# The genetical theory of multilevel selection

**DOI:** 10.1111/jeb.12566

**Published:** 2015-01-06

**Authors:** A Gardner

**Affiliations:** School of Biology, University of St AndrewsSt Andrews, UK

**Keywords:** class structure, covariance, emergence, evolutionary genetics, group selection, natural selection, Price's theorem, reproductive value, Simpson's paradox, social evolution

## Abstract

The theory of multilevel selection (MLS) is beset with conceptual difficulties. Although it is widely agreed that covariance between group trait and group fitness may arise in the natural world and drive a response to ‘group selection’, ambiguity exists over the precise meaning of group trait and group fitness and as to whether group selection should be defined according to changes in frequencies of different types of individual or different types of group. Moreover, the theory of MLS has failed to properly engage with the problem of class structure, which greatly limits its empirical application to, for example, social insects whose colonies are structured into separate age, sex, caste and ploidy classes. Here, I develop a genetical theory of MLS, to address these problems. I show that taking a genetical approach facilitates a decomposition of group-level traits – including reproductive success – into the separate contributions made by each constituent individual, even in the context of so-called emergence. However, I uncover a novel problem with the group-oriented approach: in many scenarios, it may not be possible to express a meaningful covariance between trait and fitness at the level of the social group, because the group's constituents belong to separate, irreconcilable classes.

## Introduction

Recent years have seen a resurgence of interest in the theory of multilevel selection (MLS: Price, 1972a; Hamilton, 1975; Sober & Wilson, 1998; Keller, 1999; Okasha, 2006; Wilson & Wilson, 2007; West *et al*., 2008; Gardner & Grafen, 2009; Leigh, 2010; Nowak *et al*., 2010; Lion *et al*., 2011; Marshall, 2011; Frank, 2012a, 2013). Having moved on from the controversy as to whether or not selection can operate at multiple levels – which was, in part, fuelled by confusing the weak notion of selection at the group level with the much stronger notion of adaptation at the group level (reviewed by Gardner & Grafen, 2009) – social evolution theorists now widely agree that a covariance between group trait and group fitness may arise in the natural world, resulting in a response to group selection.

However, MLS theory continues to be beset by conceptual difficulties (Okasha, 2006 provides an excellent review). Firstly, ambiguity exists over the precise meaning of group trait. The typical approach taken by MLS theorists is to treat this as a simple ‘aggregate’ of the traits of the group's constituent individuals, but some researchers have considered that group traits are often ‘emergent’ and may even be undefined at the individual level (Salt, 1979; Lloyd, 1988; Grantham, 1995; Okasha, 2006). Secondly, a similar ambiguity arises over the precise meaning of group fitness. Here, the typical approach is to define the group's fitness in terms of number of daughter individuals, but an alternative approach instead counts the number of daughter groups, and these approaches clearly disagree in the context of variable group size (Arnold & Fristrup, 1982; Damuth & Heisler, 1988; Sober, 1993; Okasha, 2006; Rainey & Kerr, 2011). Thirdly, there is ambiguity as to the focal level in a MLS analysis, with so-called multilevel selection 1 (MLS-1) describing change in the frequencies of different types of individual and multilevel selection 2 (MLS-2) describing change in the frequencies of different types of groups (Arnold & Fristrup, 1982; Mayo & Gilinsky, 1987; Damuth & Heisler, 1988; Okasha, 2001, 2006; Michod, 2011; Rainey & Kerr, 2011).

Moreover, MLS theory has not properly engaged with the problem of class structure; that is, when different individuals (or groups) differ in quality for nongenetic reasons (West *et al*., 2008; Gardner & Grafen, 2009; Frank, 2013; West & Gardner, 2013). The key issues that arise here are the following: first, that not all offspring are necessarily equal, so a simple count of offspring number may not adequately capture the notion of fitness; and, second, that chance associations between allele and class may drive evolutionary change that should not be confused with the action of natural selection and should be carefully separated out of any theoretical or empirical measure of MLS. All real-world biological populations exhibit class structure, and although neglecting such differences in quality may be reasonable for some taxa (e.g. bacteria; but see Gardner & Kümmerli, 2008), such complexity is fundamental to the biology of many organisms of social evolutionary importance. For example, within colonies of eusocial insects – the classic ‘superorganisms’ – individuals may be structured into separate age, sex, caste and ploidy classes (Gardner & Grafen, 2009). And, indeed, class structure is central to social evolutionary topics such as sex allocation (West, 2009), in which parents are judged according to the sex rather than simply the number of their offspring. Accordingly, failure to engage with class structure greatly limits the current empirical reach of MLS theory.

Here, I develop a genetical theory of MLS to address these problems. First, I describe the general theory of selection as it occurs in any medium, captured by Price's (1972a) covariance equation, and I discuss the key conceptual elements of the selection covariance. Second, I provide an overview of Fisher's (1918, 1930) genetical theory of natural selection, including the fundamental theorem of natural selection (Fisher, 1930, 1941), and I describe the action of natural selection in the context of class structure. Third, I develop an analogous genetical theory of MLS, including a fundamental theorem of MLS and a description of the action of MLS in class-structured populations. Fourth, I apply the genetical theory of MLS to resolve the definition of group trait and group reproductive success, abolish the distinction between MLS-1 and MLS-2, clarify the relationship between MLS and ‘Simpson's paradox’ (Simpson, 1951; Blyth, 1972), and identify scenarios in which the group may validly be considered a unit of selection.

## A general theory of selection

A general theory of selection is provided by Price's (1970, 1972a, 1995) theorem. In general terms, Price's theorem describes a difference between two assemblages in the average of some numerical quantity of interest. In evolutionary applications, the two assemblages are typically two generations of the same biological population and the difference between these two generations defines an evolutionary change. But Price's theorem also has applications beyond evolutionary biology (Gardner, 2008).

Price's theorem emerges from a mapping of ‘parents’ to ‘offspring’ between the two assemblages, and it decomposes the change in the average of the focal quantity into two parts: (i) ‘selection’, being the change that is due to different parents having different numbers of offspring; and (ii) ‘transmission’, being the change that is due to offspring not perfectly resembling their parents (Frank, 1995, 1998, 2012b; Price, 1995). In particular, Price's theorem captures the action of selection in a covariance form:


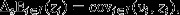
(1)

where *v*_*i*_ denotes the *i*th parent's relative contribution to the offspring assemblage (i.e. its number of offspring divided by the average number of offspring per parent) and *z*_*i*_ denotes this parent's character value (see Appendix  for details).

Price's covariance expression highlights four key conceptual elements of selection. First, the entity upon which selection acts, identified here as the holder of the index *i*, defines the ‘unit of selection’. Second, the assemblage within which selection acts, identified here by the index set *I*, defines the ‘arena of selection’. Third, the numerical property of the units, identified here as the variable *z*, whose aggregate change may be driven by selection, defines the ‘character under selection’. Fourth, the numerical property of the units, identified here as the variable *v*, which provides the measure of a unit's success, defines the ‘target of selection’. Bringing these elements together, the action of selection is given by the covariance, taken over all units within the arena, between the character and the target of selection (Appendix 1).

## The genetical theory of natural selection

### Natural selection

Natural selection is a particular kind of selection, defined by the conjunction of a particular unit, arena, character and target. Conventionally, the unit of natural selection is the individual organism, and the arena of natural selection is a biological population (Darwin, 1859). The character under selection is the heritable portion of the individual's phenotypic trait, *g*; that is, a weighted sum of the frequencies of the alleles that the individual carries, the weights being decided by linear regression analysis (Fisher, 1918; Price, 1970). This quantity is also known as the individual's ‘breeding value’ (Falconer, 1981). And the target of natural selection is the individual's ‘fitness’, *v*; that is an expectation over future uncertainty of number of offspring expressed relative to the population average (Grafen, 2000; Appendix 1). Here, I am assuming that there is no class structure, so that all offspring can be considered of equal value, but I will relax this assumption in a later section.

Making this choice of arena, unit, character and target of selection explicit in eqn 1 yields a mathematical statement of natural selection:


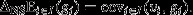
(2)

That is, the action of natural selection is given by the covariance, taken over all individuals within the population, between the individual's heritable trait and her fitness. Equation 2 has been termed the ‘secondary theorem of natural selection’ (Robertson, 1968), and I will use this term to distinguish the result specific to the action of natural selection from Price's (1972a) more general selection covariance, described in eqn 1, which has much wider application.

### The fundamental theorem of natural selection

The secondary theorem describes the action of natural selection with respect to any genetical character of interest. Perhaps the most interesting genetical character is the heritable component of fitness itself (Fisher, 1941). Fitness may be decomposed into its genetical and environmental components, that is *v*_*i*_ = *g*_*i*_ + *e*_*i*_, where *e*_*i*_ captures nonadditive genotypic effects (such as dominance, epistasis, synergy and frequency dependence) as well as other more obviously environmental effects. Making this substitution into eqn 2 yields 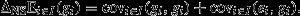 And, as 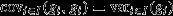 and 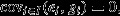 this obtains the ‘fundamental theorem of natural selection’:



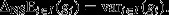
(3)

That is, the change in average fitness ascribed to the action of natural selection is equal to the (additive) genetic variance in fitness (Fisher, 1930, 1941). The importance of this result is that, because variances are nonnegative, natural selection can only have an improving effect on fitness. Fisher (1930) used the fundamental theorem as justification for the idea that individuals will appear designed to maximize their fitness (see Grafen, 2002, 2003 for more on this optimization view).

Importantly, the fundamental theorem is not concerned with total evolutionary change in fitness, but only the action of natural selection (Price, 1972b). Nonselective change in fitness owing to mutation and changing associations between genes and fitness – collectively termed ‘deterioration of the environment’ by Fisher (1930) – tends to reduce average fitness (Frank & Slatkin, 1992). In the past, this subtlety has been lost on many evolutionary theorists who, considering the fundamental theorem to be a statement about total evolutionary change in fitness, asserted that it is incorrect or only applies under very special conditions (reviewed by Edwards, 1994). This conceptual confusion illustrates the importance of being able to mathematically separate the selective versus nonselective components of evolutionary change (Appendix 1).

Today, disagreement still persists as to the correct interpretation of the fundamental theorem. For example, whereas Okasha (2008) and Ewens (2011) both regard the theorem as concerning the selection of genes, I regard it as concerning the selection of individuals. Although the fundamental theorem describes change in a genetical character, this change is driven by the differential fitness of individuals. Moreover, the genetical character represents information – carried by genes – about the fitness of individuals. That is, the fundamental theorem emerges from a selection covariance in which the unit of selection is the individual, the target of selection is the individual's fitness, and the character under selection is the heritable portion of the individual's fitness. Here, genes merely provide a material basis for the inheritance of the individual's character. Indeed, as the above derivation applies equally well to blending inheritance, genes cannot play a key role in the theorem's logic (cf. Gardner, 2011). These points illustrate the importance of being able to conceptually separate the unit, arena, character and target of selection.

### Natural selection in class-structured populations

If individuals vary in their propensity to achieve reproductive success, for reasons other than the genes that they carry (e.g. owing to differences in age, sex, caste and/or local habitat; Grafen, 2006), then natural selection cannot be described as a simple covariance of genetic value with fitness, taken across all individuals in the population. Firstly, spurious correlations between heritable traits and nongenetic aspects of individual quality may drive genetic changes that should not be conflated with the action of natural selection (Taylor, 1990). Secondly, if offspring vary systematically in their propensity to achieve reproductive success, then a simple count of offspring number need not capture an individual's genetic legacy across multiple generations (Price & Smith, 1972). A solution to this problem is to: (i) separate individuals into classes, such that the only differences within classes are genetical in nature; (ii) describe the action of natural selection separately for each class; and (iii) compute the overall action of natural selection as a sum across all classes, giving each class a weight according to the neutral expectation of its long-term genetic contribution to future generations (i.e. its ‘reproductive value’; Fisher, 1930; Price, 1970; Price & Smith, 1972; Taylor, 1990). This yields:


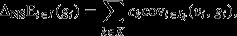
(4)where *I*_*k*_ denotes the subset of the index set *I* pertaining to the *k*th class, *c*_*k*_ is the reproductive value of the *k*th class, and relative fitness *v*_*i*_ is expected offspring number divided by the average for all individuals of that class (see Appendix 2 for details).

The basic idea here is that, in a class-structured population, an allele's frequency may undergo systematic change even if that allele is entirely neutral. Accordingly, even if natural selection is playing some role in driving allele frequency change, it may not be responsible for all of this change. And so, to properly describe the action of natural selection in terms of genetical change, it is important to: consider a counterfactual scenario in which alleles are neutral and remain that way until the end of time; determine the corresponding change in their frequencies under neutrality; and then subtract this from the actual allele frequency change that occurs in the real-world scenario in which natural selection is operating. The class reproductive values describe the expected genetic contribution that each class makes to the distant future in the neutral counterfactual scenario. Hence, they are calculated under the assumption of neutrality, even though the wider context is one in which the action of natural selection is being described (see the ‘Class effects and Simpson's paradox’ section, below, for more discussion).

In the context of class structure, natural selection is given by the class-reproductive-value-weighted sum (taken over all classes) of the covariance (taken over all individuals within a class) between the individual's heritable trait and her fitness. Accordingly, the arena of each selection covariance is the subpopulation of individuals belonging to a particular class. This is the approach taken by Price (1970), and my eqn 4 can be seen as a generalization of his eqn 5, which focused specifically upon populations structured into female versus male classes and X-linked genes.

## The genetical theory of MLS

### Multilevel selection

In the context of social evolution, in which social interaction between individuals mediates the covariance of fitness and genetic values, it is often helpful to decompose the overall response to natural selection into separate parts, to aid conceptualization (Gardner *et al*., 2007). The MLS approach separates natural selection into its within-group versus between-group components (Price, 1972a; Hamilton, 1975; Okasha, 2006). Assuming the absence of class structure, assigning every group a unique index *j* ∈ *J*, assigning each individual to a single group and denoting the subset of the population that comprises the *j*th group by *I*_*j*_, eqn 2 may be rewritten as follows:


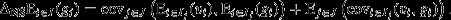
(5)

The right-hand side of eqn 5 expresses the action of natural selection as the sum of two terms. The first of these terms is a selection covariance, in which the unit of selection is the group (indicated by the index *j*), the arena of selection is the population of groups (indicated by the index set *J*), the character under selection is the average genetic value among the individuals in the group (denoted 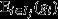), and the target of selection is the average fitness among the individuals in the group (denoted 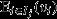). This selection covariance describes selection that is operating at the between-group level, and provides a formal definition of ‘group selection’ (Price, 1972a; Hamilton, 1975). Here, the target of group selection – that is the average fitness among the individuals in the group – provides an operational definition for ‘group fitness’.

The second term is an expectation of selection covariances, in which the unit of selection is the individual (indicated by the index *i*), the arena of selection is the set of individuals within a particular group (indicated by the index set *I*_*j*_), the character under selection is the individual's genetic value (denoted *g*_*i*_), and the target of selection is the individual's relative fitness (denoted *v*_*i*_). This selection covariance describes selection operating at the within-group level, and its expectation across all the groups in the population defines ‘within-group selection’ (Price, 1972a; Hamilton, 1975). Note that, as the unit of selection here is the individual, within-group selection has some conceptual claim on the term ‘individual selection’. However, the same logic would lead to the RHS of eqn 2 also being termed ‘individual selection’. To avoid such ambiguous language, I instead use ‘within-group selection’ to describe the term in eqn 5 and ‘natural selection’ to describe the term in eqn 2.

### The fundamental theorem of MLS

Equation 5 might be termed the ‘secondary theorem of MLS’, in analogy with eqn 2. This suggests the possibility for a ‘fundamental theorem of MLS’. Taking group fitness 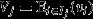 as the character of interest, and assigning this a genetic component 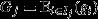 and an environmental component E_*j*_ in the usual way, yields 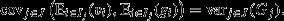 Noting that Δ_NS_E_*i*∈*I*_(*g*_*j*_) = Δ_NS_E_*j*∈*J*_(*G*_*j*_); then, from eqn 5:


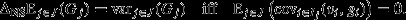
(6)

That is, the change in average group fitness owing to the action of natural selection is equal to the genetic variance in group fitness if and only if there is no selection within groups. This provides an informal proof of the idea that groups will only appear designed to maximize their fitness if there are mechanisms – such as clonality or repression of competition – that more-or-less totally abolish selection within groups; otherwise, natural selection may favour traits that decrease group fitness and disfavour traits that increase group fitness (see Gardner & Grafen, 2009; Gardner, 2013 for more on this optimization view).

### MLS in class-structured populations

Equation 4 provides an expression for the action of natural selection in a class-structured population. Assigning individuals to groups *j* ∈ *J*, and applying the MLS partition


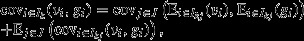
separately for each class obtains:


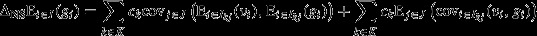
(7)which separates the action of natural selection into between-group and within-group components. Note that the between-group selection covariances have, as their unit of selection, not a whole social group, but rather the subgroup of individuals in each social group that belong to the same class. Correspondingly, the target of between-group selection is the average fitness of individuals within the pure-class subgroup, the character under between-group selection is the average heritable trait of the individuals within the pure-class subgroup, and the arena of between-group selection is the population of pure-class subgroups belonging to the same class. Similarly, the within-group selection covariances have the individual as the unit of within-group selection, the individual's heritable trait as the character under within-group selection, the individual's fitness as the target of within-group selection and the pure-class subgroup as the arena of within-group selection.

## Class effects and Simpson's paradox

In eqn 7, I have described the action of MLS in a class-structured population, controlling for spurious correlations between heritable traits and fitness that may arise when individuals vary in quality for other reasons, and that should not be mistaken for the action of MLS itself. The idea here is that calculating a covariance is mathematically analogous to performing a least-squares regression analysis (Gardner *et al*., 2011) and so, by calculating selection covariances separately for each class, the effects of any confounding variables – that collectively define class membership – are removed. Moreover, weighting each selection covariance by the reproductive value of the corresponding class describes its long-term genetic impact upon the population, and the sum of the weighted selection covariances describes the overall action of MLS.

For example, consider a neutral or weakly deleterious allele that is lucky enough to find itself overrepresented among high-quality individuals. The overall correlation between gene and fitness may be positive, because carriers of the allele tend to be fitter than noncarriers for reasons that have nothing to do with them carrying the allele. Accordingly, in the absence of other evolutionary forces, the allele will increase in frequency, in an apparent contradiction of Darwin's (1859) remark: ‘This preservation of favourable variations and the rejection of injurious variations, I call Natural Selection. Variations neither useful nor injurious would not be affected by natural selection’. The apparent contradiction is resolved by noting that this change in allele frequency is not natural selection, but rather a distinct ‘class effect’. To be clear, the class effect is not particular to MLS and may also arise in the context of kin selection analysis: naïve application of covariance (or least-squares regression or differentiation) methodology is liable to give nonsensical results in the context of class structure (Allen *et al*., 2013). Taylor (1990) and Taylor & Frank (1996) give excellent accounts of kin selection analysis for class-structured populations.

The class effect relates to a statistical phenomenon known as ‘Simpson's paradox’ (Simpson, 1951; Blyth 1972), in which the association between two variables disappears or even reverses when a third, confounding, variable is controlled for. The paradox arises when correlation is interpreted as straightforward causation, such that the same dataset yields two mutually incompatible causal interpretations (Pearl, 2009, 2014). A classic example relates to a case of apparent sex discrimination in the admission of graduate students to the University of California at Berkeley: female applicants were much less likely to be admitted than their male counterparts, suggesting discrimination against women, when all admissions were considered as a whole; yet, this pattern vanished when admissions to each department were considered individually, suggesting no such discrimination was occurring (Bickel *et al*., 1975). Further analysis revealed that female applicants tended to apply to departments where overall rates of admission were lower, which explained the apparent sex bias, and the University was exonerated (Bickel *et al*., 1975).

Simpson's paradox has previously been discussed in the MLS literature, although not in relation to the confounding effects of class. In fact, it has been used to describe the action of MLS itself. Sober & Wilson (1998) have drawn an analogy between a group-structured population, in which altruism is associated with reduced fitness within every group but higher fitness within the population as a whole, on the one hand, and the Berkeley sex discrimination case, on the other. However, I believe that this is a poor analogy. Altruism, in Sober & Wilson's (1998) model, is associated with higher fitness overall, not because of any confounding variable, but rather because of the causal action of altruism itself: groups of altruists are fitter because they are groups of altruists. This is very different from the Berkeley case, in which the low rates of admission to certain departments were – supposedly – not due to their attracting mainly female applicants. Indeed, if Berkeley had been deliberately allocating fewer graduate student positions to these departments because they were popular with women, then this would clearly have been sex discrimination.

## Collective fitness_1_ versus collective fitness_2_

A much-discussed problem with the theory of MLS is that it has not been clear whether a group's reproductive success should be defined in terms of its number of daughter individuals or its number of daughter groups (Arnold & Fristrup, 1982; Damuth & Heisler, 1988; Sober, 1993; Okasha, 2006; Rainey & Kerr, 2011). This clearly matters when there is variation in group size. Okasha (2006) provides an illustrative example, in which group A produces twelve daughter individuals organized into four groups of three and group B produces twelve daughter individuals organized into three groups of four. By what he terms ‘collective fitness_1_’, which counts the number of daughter individuals, groups A and B are equally successful. But, by what he terms ‘collective fitness_2_’, which counts the number of daughter groups, group A is more successful than group B.

The genetical theory of MLS provides a solution to this problem, by defining the reproductive success of any unit in terms of its expected long-term genetic contribution to future generations. Because the reproductive value of any group is a simple sum of the reproductive values of its constituent individuals, the reproductive value of the mother group can be calculated either as the sum of the reproductive values of its daughter individuals or as the sum of the reproductive values of its daughter groups, and these two calculations will always yield the same answer.

In an empirical context, simply counting the number of daughter individuals – that is the collective fitness_1_ approach – is appropriate when there is negligible class structuring of individuals. Such a scenario is unlikely when groups vary in size and individuals engage in social interactions within their groups, as individuals in differently sized groups will experience rather different social environments, even in a genetically homogenous population. Conversely, simply counting the number of daughter groups – that is the collective fitness_2_ approach – is appropriate when there is negligible class structuring of groups. Such a scenario is also unlikely when groups vary in size, unless there is extreme density regulation such that small groups achieve the same overall productivity as large groups. More generally, even though the collective fitness_1_ and collective fitness_2_ approaches will converge upon the same measure of reproductive success in the absence of variation in group size, this measure may nevertheless be inadequate if individuals and groups are class structured in other ways. Daughter individuals or groups of low quality should not be given the same weight as daughter individuals or groups of high quality in computing the reproductive success of the parent group but, instead, each daughter individual or group should be weighted in proportion to its reproductive value.

Viewing reproductive value as a proper measure of an entity's evolutionary success clarifies the relationship between cancer and MLS. Cancer is often conceptualized as involving a tension between different levels of selection, with cancerous tissues achieving higher reproductive success at a within-organism level and cancerous individuals suffering lower reproductive success at a between-organism level (Okasha, 2006; Clarke, 2011; Foster, 2011; Goodnight, 2013). However, somatic tissues – including cancerous ones – do not generally contribute genes to distant future generations, on account of the demise of their lineages upon the death of the organism (Clarke, 2011; Goodnight, 2013). Consequently, cancerous tissues do not have reproductive value, and so their proliferation within the organism cannot correspond to selection in the strict sense of the genetical theory. The exception is transmissible cancer – such as that causing devil facial tumour disease in Tasmanian devils, and transmitted by biting (Pearse & Swift, 2006) – which has the potential to survive indefinitely and hence achieve reproductive value. In such cases, the cancer represents a separate, parasitic individual – perhaps even belonging to a distinct species (cf Vincent, 2010) – rather than a rebellion of the host individual's own tissues.

## Aggregate characters versus emergent characters

Analogous to the apparent problems that have been posed in the literature concerning group fitness, there has been much discussion of how best to conceptualize group-level traits (Salt, 1979; Lloyd, 1988; Grantham, 1995; Okasha, 2006). Adopting Okasha's (2006) terminology: the majority of MLS models have considered ‘aggregate’ traits, where the group trait value is a simple average of the trait values of its constituent individuals; but this approach has been regarded as incapable of capturing the action of MLS when group traits are ‘emergent’ and perhaps even undefined at the individual level. Okasha (2006) discusses the example of the group's sex ratio, which is a property of the group rather than of any of its constituent individuals (although he notes that each individual in the group does have the individual-level property of being in a group with that sex ratio).

The genetical theory of MLS resolves this problem by considering that natural selection acts only upon the heritable portion of the phenotype; that is, the character under selection is strictly genetical. Importantly, any biological entity that contains genes may be ascribed a trait value that is a simple weighted sum of the frequencies of the various alleles that it carries, irrespective of whether that entity is an individual or a group. And the genetical character may relate to a phenotype that is expressed at any level of biological organization, not necessarily the one occupied by the focal entity.

The genetical approach is entirely consistent with the ‘aggregate’ view of group-level traits, in that the group's genetical trait value is a simple weighted sum of the genetical trait values of its constituent individuals. But it is also entirely consistent with the ‘emergent’ view of group-level phenotypes, which do not need to be defined at an individual level in order for individuals to be assigned genetical scores for them. This is analogous to how a bull may be assigned a breeding value for milk yield, as a function of his genotype, even though he does not have udders. Such assignment is neither arbitrary nor anomalous, but rather plays an important role in the practice of artificial selection, because bulls carry genes for milk yield and pass them on to their daughters, who do express them. Similarly, in a social evolutionary context, natural selection for the phenotypes of sterile insect workers is driven by the differential fitness of reproductive individuals who have heritable predispositions for, but do not actually exhibit, those phenotypes (Darwin, 1859).

## MLS-1 versus MLS-2

In addition to the difficulties associated with group-level fitness and group-level traits, the literature on MLS has been much concerned with the question of how to describe the evolutionary change associated with group selection. Building upon the ideas of Damuth & Heisler (1988), Okasha (2006) distinguishes ‘MLS-1’, which describes change in the frequencies of different types of individual (or, more generally, different types of ‘particle’), versus ‘MLS-2’, which describes change in the frequencies of different types of group (or, more generally, different types of ‘collective’; see also Arnold & Fristrup, 1982; Mayo & Gilinsky, 1987; Okasha, 2001). Michod (2011) and Rainey & Kerr (2011) discuss the MLS-1 versus MLS-2 distinction in the context of major transitions in evolution (Maynard Smith & Szathmáry, 1995).

The genetical theory of MLS adopts neither of these two approaches and, instead, describes the action of group selection in terms of change in a genetical character. As discussed in the previous section, a genetical score may be assigned to any biological entity that contains genes – such as an entire population – and change in this genetical score can be computed, irrespective of how that population is subdivided into groups and individuals, or the biological level of organization at which the corresponding phenotype actually manifests.

One might argue that this genetical approach is merely an extended MLS view that considers a lower tier of particles – the genes – and that this is therefore a form of MLS-1. However, this is incorrect, for two reasons. Firstly, describing change in the average value of a genetical character is not equivalent to describing change in the frequencies genetic types. Rather, the genetical character describes an arbitrarily weighted sum of potentially multiple allele frequencies, and although these frequencies determine the value of the genetical character, the reverse need not be true. Secondly, the basic selection covariance logic can also be applied to heritable characters that do not have a particulate basis (i.e. blending inheritance; Gardner, 2011). This clarifies the sense in which the theory of natural selection is ‘genetical’: this adjective pertains to the medium by which characters are inherited, rather than to the unit of selection itself.

## Are social groups units of selection?

In eqn 7, I decomposed the action of natural selection in a class-structured population into separate between-group and within-group components. Here, the component of natural selection that is occurring between groups is given by:


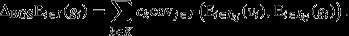
(8)

In contrast to the corresponding term appearing in eqn 5, which described the MLS partition in the absence of class structure, this quantity is not readily interpretable as a selection covariance in which the whole group acts as a unit of selection. Instead, it is a reproductive-value-weighted sum of selection covariances, each taken over different pure-class subgroups of individuals rather than over entire social groups. Accordingly, it is the pure-class subgroup, not the entire social group, that acts as the unit of selection.

This raises the question of whether and when a whole social group can be considered a viable unit of selection, with some measure of group fitness providing the target of group selection, and some measure of group genetic value providing the character under group selection. This can be shown to obtain in some special scenarios. First, if every social group is homogeneous with respect to class, then the pure-class subgroup is synonymous with the social group itself, and hence, from eqn 8, the social group is a unit of selection, its fitness 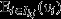 is the target of group selection, and its genetic value 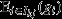 is the character under group selection. A trivial example of when this scenario will apply is when the whole population lacks class structure, as assumed, for example, by the models of Gardner & Grafen (2009). However, the scenario will also apply to class-structured populations so long as all class differences are between rather than within groups, as assumed, for example, by the models of Rodrigues & Gardner (2012), that consider variation in resource availability among different groups.

Second, if the pure-class subgroups of a social group are constrained to have the same average genetic values (i.e. 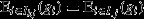 for all *j* ∈ *J* and all *k* ∈ *K*, where *I*_•*j*_ is the set of all individuals within the *j*th group), then eqn 8 may be re-expressed as 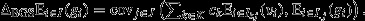 This recovers the interpretation of the entire social group as a unit of selection, with a reproductive-value-weighted average of the fitnesses of its constituent pure-class subgroups 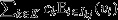 providing the target of group selection and its genetic value 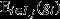 providing the character under group selection. One example of when this scenario will apply is when all of a group's constituent individuals are genetically identical (i.e. 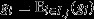 for all *i* ∈ *I*_*.j*_). Biologically, such group clonality appears to be the most plausible mechanism for ensuring that the pure-class subgroups have the same genetic values, but the former is not strictly required for the latter to obtain.

Third, if the fitnesses of all of a social group's pure-class subgroups are equal (i.e. 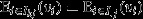 for all *j* ∈ *J* and all *k *∈ *K*), then eqn 8 may be re-expressed as 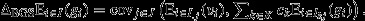 This recovers the interpretation of the entire social group as a unit of selection, with the fitness of the social group 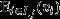 providing the target of group selection and a reproductive-value-weighted average of the genetic values of its pure-class subgroups 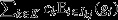 providing the character under group selection. Note that this scenario does not require that all pure-class subgroups have equal absolute reproductive success, but rather that their relative reproductive success (i.e. absolute offspring number divided by the average for their class) is equal for all subgroups within the social group. Moreover, it also allows for fitness variation within the pure-class subgroups.

The issue of whether a group can be considered a unit of selection is distinct from that of whether a group can be considered a unit of adaptation, that is a fitness-maximizing entity. The former requires that a nonzero portion of natural selection can be expressed as a selection covariance in which the social group plays the role of unit of selection and may be assigned a meaningful measure of fitness. The latter has the additional requirement that there is also zero selection within groups – as shown in expression 6 and by Gardner & Grafen (2009) – such that the necessary and sufficient criterion for any heritable trait to be favoured by natural selection is that it improves group fitness.

The importance of being able to describe a selection covariance that identifies the whole social group – and not simply the pure-class subgroup – as a unit of selection is made vivid by considering scenarios in which no two individuals in the same social group belong to the same class and in which neither the genetic uniformity nor the relative fitness uniformity criteria are satisfied. For example, a parasitoid wasp might oviposit a single unfertilized (i.e. male) egg and a single fertilized (i.e. female) egg into a caterpillar, within which these siblings develop and compete for resources, and this yields both a clearly defined social group of more than one individual and also ample scope for kin selection. Yet, it is unclear whether group selection can occur, except in the trivial sense that a single individual can be considered a group of size 1, owing to difficulties in bringing the separate selection covariances for male subgroups and for female subgroups together into a single selection covariance.

From a conceptual perspective, this point may help to illustrate the more general point that, although kin selection and MLS methodologies are equivalent (they both describe the action of natural selection, and simply carve it up in different ways), kin selection is not a special kind of group selection that operates between kin groups (contra Wilson, 1975). Indeed, there can be kin selection in the absence of group selection, as defined above, even in populations that are structured into clearly defined kin groups. From an empirical perspective, this point highlights that the total reproductive success of a heterogeneous group may be a meaningless quantity and that scientific resources might be more profitably invested into measuring other things.

## Conclusion

A genetical approach to MLS addresses several of the difficulties that have beset this theory of social evolution. Here, I have resolved the meaning of group trait and group fitness, highlighted that MLS is defined by change in a genetical character driven by its covariance with fitness at individual and group levels and clarified the connection between MLS and Simpson's paradox. Moreover, by integrating the theories of class structure and reproductive value, I have extended the empirical reach of MLS theory. However, these developments have shown that it may not always possible to treat whole social groups as units of selection and that often separate gene-fitness covariances must be taken over pure-class subgroups instead. For many empirical scenarios in which social groups comprise individuals of more than one class, it may not be possible to bring together the between-group components of within-class selection into a single conception of ‘group selection’, even in the context of kin selection and social evolution.

## Figures and Tables

**Figure 1:**
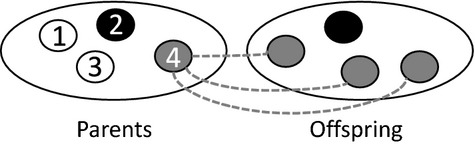
The mapping that forms the basis of Price's equation. Differences in shading represent differences in character value, and the Price equation describes change in the average character value between parent and offspring populations.

**Figure 2:**
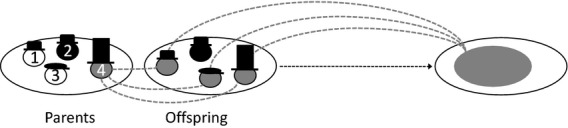
The Price equation mapping for a class-structured population. When individuals differ both in their genetical characters (shading) and in their class (hats), number of offspring does not provide an adequate measure of evolutionary success, i.e. long-term contribution of genes to future generations.

**Figure 3:**
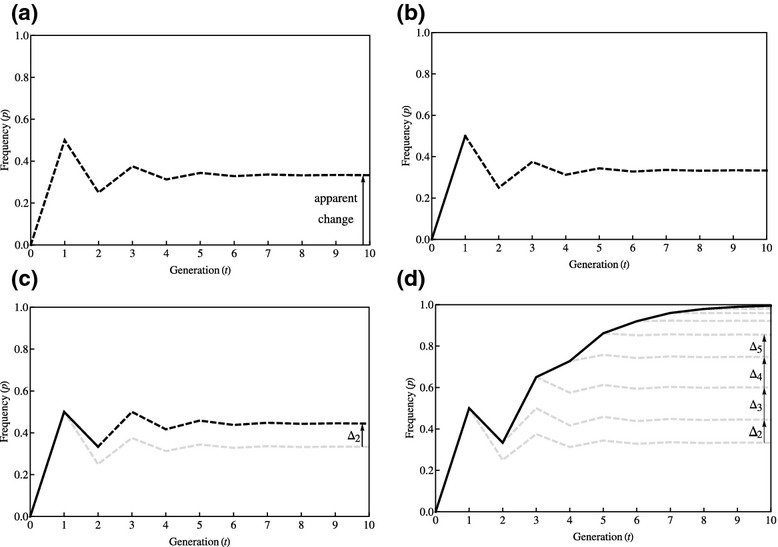
Dynamics of allele frequency change under haplodiploidy. Here, *△*_*t*_ describes the portion of the expected long-term genetic change that owes to the action of natural selection in generation *t*.

**Figure 4:**
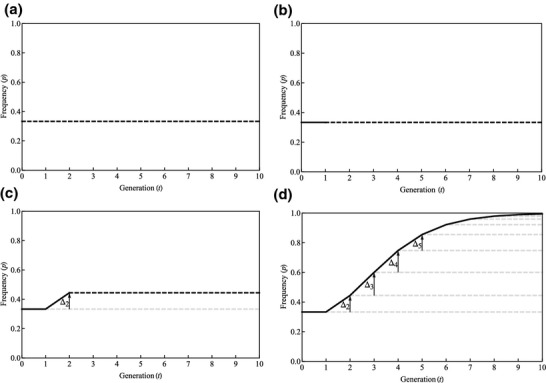
Dynamics of class-reproductive-value-weighted allele frequency change under haplodiploidy. Here, *△*_*t*_ describes the reproductive-value-weighted expected short-term genetic change that owes to the action of natural selection in generation *t*, and which is conceptually different – but numerically equivalent – to how natural selection is captured in Figure3.

## Appendix 1

### The Price equation

Price's (1970, 1972a) theorem emerges from a mapping between two assemblages of entities – a ‘parent’ assemblage and an ‘offspring’ assemblage – which need not be of a biological nature (Figure1). Each of the entities in the parent assemblage is assigned a unique index *i* ∈ *I*, and its absolute number of descendants in the offspring assemblage is denoted by *w*_*i*_. The arithmetic average of *w*_*i*_ among all the entities in the parent assemblage is E_*i*∈*I*_(*w*_*i*_) = ∑_*i*∈*I*_*q*_*i*_*w*_*i*_,  where equal weighting is given to each parent, that is *q*_*i*_ = *q* for all *i* ∈ *I* and ∑_*i*∈*I*_*q*_*i*_ = 1. Thus, each parent's success may be expressed in a relative way, as *v*_*i*_ = *w*_*i*_/E_*l*∈*I*_(*w*_*l*_). The parents may be scored for any property of interest, and accordingly, each is assigned a numerical trait score *z*_*i*_, and the average trait value in the parent assemblage is E_*i*∈*I*_(*z*_*i*_) = ∑_*i*∈*I*_*q*_*i*_*z*_*i*_. Finally, a parent's descendants are collectively assigned an average trait value  where Δ*z*_*i*_ captures the difference between parent and offspring trait values, and the average trait value in the entire offspring assemblage is

Hence, the total change in the average trait value between parent and offspring assemblages is  or:

(A1 1)

where E denotes an arithmetic average or expectation and cov denotes a covariance, each taken over the indicated set (Price, 1972a). The left-hand side of eqn 9 denotes the change in the population average of the character. The right-hand side of eqn 9 expresses this change as the sum of two terms. The first term is the change ascribed to selection and is equal to the covariance of relative success and character value, across all entities in the parent population. The second term is the change ascribed to transmission and is equal to the average (relative-success-weighted) difference between the character values of a parent and its offspring.

In some applications of Price's theorem, there is not one offspring assemblage, but rather a set of possible offspring assemblages, each having some probability of realization. Assigning each possible offspring assemblage a unique index *ω* ∈ *Ω* and denoting parent *i*'s relative contribution of offspring under realization *ω* by  where  is parent *i*'s absolute contribution of offspring under realization *ω*, eqn 9 may be rewritten as follows:

(A1 2)

which describes the character transformation in the event of realization of offspring assemblage *ω*. In such applications of Price's theorem, it is often appropriate to describe the expected change, averaging over uncertainty as to which of the offspring assemblages will be realized. This is given by:

(A1 3)

However, this notation is a bit cumbersome, and it is often more convenient to leave the expectation over uncertainty implicit. See Grafen (2000) and Gardner & Grafen (2009) for more on expectations over uncertainty in the context of Price's theorem.

Price's theorem is a mathematical tautology, arising from simple notational definitions rather than from mechanistic assumptions. Consequently, it is not very useful for making concrete predictions about evolutionary change. Instead, its usefulness lies in how it provides general definitions for components of evolutionary change. In particular, Price's theorem provides a general, formal definition of selection: isolating the first term from the right-hand side of eqn 9 recovers eqn 1 of the main text.

Moreover, Price's theorem highlights four key conceptual elements of selection: selection is defined in terms of change in the expectation of a random variable *z*, and this variable formally defines the ‘character under selection’; selective change in the character is equal to its covariance with a second random variable *v*, and this variable formally defines the ‘target of selection’; these random variables are themselves formally defined by drawing entities at random from an aggregate and noting their associated character and target values (Gardner *et al*., 2011, box 1), the entities being drawn formally defining the ‘unit of selection’ and the aggregate from which they are drawn formally defining the ‘arena of selection’.

### Appendix 2

#### Natural selection in class-structured populations

The action of natural selection in the absence of class structure is given by eqn 2 of the main text. Here, I derive an expression for the action of natural selection in the presence of class structure, namely eqn 4 of the main text. Accordingly, I assign individuals to classes, such that all the individuals in the same class have the same nongenetic quality. Specifically, in addition to assigning every individual a unique index *i *∈ *I*, I assign every class a unique index *k* ∈ *K*. The subset of individuals belonging to class *k* is denoted *I*_*k*_ (Figure2).

Equation 2 of the main text was derived from a mapping between consecutive parent and offspring generations, and this is appropriate in the absence of class structure because all offspring have equal value, and hence, expected relative contribution of offspring to the next generation provides a proper measure of each parent's evolutionary success. However, in the context of class structure, offspring may vary in their value, and it is necessary to instead consider each individual's expected long-term genetic contribution to future generations; that is, her ‘reproductive value’ (Figure2). I denote individual *i*'s reproductive value as *f*_*i*_ and, following Taylor (1990), I scale this such that the average reproductive value among all the parent individuals is E_*i*∈*I*_(*f*_*i*_) = 1. Note that other scalings are equally valid: for example, figure 2 of Fisher (1930) employed a scaling such that a female's reproductive value at birth is 2.

The selection covariance emerging from this mapping between the parent generation and a distant future generation is  Note that this is analogous to the selection covariance on the RHS of eqn 2 of the main text, except that the target of selection is the individual's expected long-term genetic contribution to the future (reproductive value, *f*_*i*_) rather than the individual's expected relative offspring number (fitness, *v*_*i*_). However,  does not provide a proper account of the action of natural selection acting in the parental generation, because it includes effects of class membership (i.e. because individuals vary in quality for nongenetic reasons,  may be nonzero even in a neutral population in which natural selection cannot be acting), and because it includes the effects of natural selection in all generations from the present into the distant future. These separate effects may be isolated by writing  where  is the reproductive value that the *i*th individual would enjoy under neutrality and Δ_*t*_*f*_*i*_ is the deviation from this neutral expectation owing to gene effects in the *t*th generation, starting with her own generation at *t *= 1.

That is, if, in addition to considering the real-world scenario in which selection is operating, one considers a counterfactual scenario in which all genes are neutral, *f*_*i*_ describes the individual's expected long-term genetic contribution in the selection scenario,  describes her expected long-term genetic contribution in the neutral counterfactual scenario, and the difference between these two quantities  describes the cumulative action of natural selection acting in every generation from the present to the distant future. Thus, there are three ways for individuals to achieve high reproductive value: they may be born with high reproductive value, on account of their class (high ); they may achieve high reproductive value, on account of the action of their superior genes (high Δ_1_*f*); and they may have high reproductive value thrust upon them on account of the genetic superiority of their descendants (high ).

To make this decomposition of reproductive value more concrete, I write , where *v*_*i*_ is the individual's expected number of offspring relative to the average for her class and  is the average reproductive value she gains for each of these standardized offspring units, in the selection scenario. Expected relative number of offspring may be written as  where  is the expectation under the neutral counterfactual scenario and Δ*v*_*i*_ is the deviation in expected relative number of offspring owing to natural selection. Likewise, average reproductive value per standardized offspring unit may be written as  where  is the expectation under the neutral counterfactual scenario and  is the deviation due to natural selection. It follows that  and

Substituting the components of reproductive value into the selection covariance  yields:

(A2 1)

The first term on the RHS of eqn 12 describes the portion of the expected long-term genetic change that would occur even if the genes were entirely neutral in their effects, which I term the class effect. The second term describes the portion of the expected long-term change that occurs because of the impact of genes on fitness in the focal generation, which defines the immediate action of natural selection. And the third component describes the portion of the expected long-term change that occurs because of the impact of genes on fitness in future generations. An illustrative example of this partition of class and selective effects is given in Appendix 3.

Hence, a proper statement of the immediate action of natural selection, acting in the present generation, but having a long-term impact upon the genetic composition of the population, is given by the second term on the RHS of eqn 12:

(A2 2)

Here, the target of natural selection is not the entirety of the individual's reproductive value, but rather the portion that owes to the impact of genes on fitness. It is defined for any strength of selection, but its conceptualization has involved making a comparison with a neutrality counterfactual scenario in which selection is absent. Note, the LHS of eqn 13 describes a portion of the actual expected long-term genetic change, and not a reproductive-value-weighted expected short-term genetic change (see below for more discussion).

Typically, the action of natural selection in the context of class structure is written as a weighted sum of covariances that are taken separately over individuals of each class: for example, eqn 5 of Price (1970). To express eqn 13 in this form, I first separate its RHS into its within-class versus between-class effects:

(A2 3)

Note that, by virtue of the definition of class, all individuals belonging to the same class have offspring with the same neutral reproductive value (which entails  for all *i *∈ *I*_*k*_ and all *k* ∈ *K*). Accordingly, , so that eqn 14 may be rewritten as follows:

(A2 4)

where  is the proportion of parental individuals that belong to the *k*th class. Finally, making the substitution  recovers eqn 4 of the main text. Here, *c*_*k*_ is the reproductive value of class *k*, being the probability that a gene drawn at random from the distant future would originate from class *k* in the present generation, were there to be no natural selection operating in the present – or any future – generation. For the special case in which all individuals belong to the same class – that is there is only one element *k* ∈ *K*, such that *I*_*k*_ = *I* and *c*_*k*_* *= 1 – eqn 4 reduces to eqn 2.

Note that the above treatment of natural selection in class-structured populations makes no assumption of weak selection or vanishingly rare mutant alleles and, accordingly, it differs in various details from some previous treatments (Taylor, 1990, 1996). Indeed, whereas the apparent contradiction of defining the action of natural selection in terms of class reproductive values that are calculated under neutrality has typically been resolved by assuming vanishingly weak selection, my resolution instead involves a contrast between a natural selection scenario and a neutral counterfactual scenario, whereby the class reproductive values emerge from consideration of the latter and are used to ascertain how much of the expected genetic change occurring in the former would have occurred even in the absence of natural selection.

Also, I have described the individual's reproductive value as the expectation over uncertainty of her genetic contribution to the distant future, and this differs from some previous uses of the term, to describe either her realized long-term contribution or her expected contribution conditional upon a given pedigree (i.e. her descendants are specified but uncertainty remains as to the genes that they carry; Barton & Etheridge, 2011). In addition, I have conceptualized natural selection as being driven by fitness differences – that is differences in expected relative offspring number – within classes. Selection for traits that alter offspring class rather than offspring number, such as sex allocation, may be conceptualized as acting upon the offspring themselves and driven by differences in their fitness, as was done by Taylor & Frank (1996). That is, such effects contribute to the action of natural selection in the subsequent, rather than the present, generation.

Finally, I have conceptualized the action of natural selection in the context of class structure as a portion of the expected long-term genetic change of the population, as opposed to the immediate genetical change occurring from one generation to the next (or a portion thereof). This differs from previous treatments, beginning with Fisher (1930), that have conceptualized the action of natural selection in terms of the immediate change in the class-reproductive-value-weighted average of the frequencies of alleles across the different classes in the population. My approach directly relates to the idea that natural selection has long-term consequences for biological populations that may not be fully captured by considering only a single generation of actual genetic change. Fisher's (1930) approach provides a convenient means of bringing those long-term effects into the focal generation, by incorporating information about the future prospects of alleles into their present population frequencies. Although conceptually distinct, these two approaches yield exactly the same mathematical result, and their numerical equivalence is illustrated in Appendix 3. A mathematical and historical overview of the theory of reproductive value, generalizing beyond discrete classes, is provided by Grafen (2006).

### Appendix 3

#### Allele frequency change in a haplodiploid population

Many animal species exhibit haplodiploid inheritance, whereby daughters are produced in the usual way, by fusion of a female's egg with a male's sperm, but males develop from unfertilized eggs. Consequently, males are haploid and females are diploid. Males draw all their genes from their mother, whereas females draw half of their genes from each parent.

This bizarre form of inheritance may lead to complicated gene frequency dynamics, comprising both class effects and truly naturally selective effects. For example, consider a haplodiploid population in which there is a strongly female biased sex ratio that remains constant over generations, so that the ‘*per capita*’ frequency of any gene at any time is approximately equal to its frequency in females (this may be unrealistic if male fecundity is limiting; Gardner, 2014). If all of the males are initially hemizygous for a neutral allele A and all of the females are initially homozygous for a neutral allele *α* at the same locus then, initially, the frequency of the A allele will be approximately zero (because males are rare). However, in the next generation the frequency of this allele will leap to approximately 0.5, because every female will inherit this allele from her father and will inherit the other allele from her mother. Moreover, none of the males in this generation will carry the A allele. Consequently, in the second generation, the frequency of the A allele will be approximately 0.25, because only half of the females will inherit it from their mother and none of them will inherit it from their father. Table1 records the allele frequencies over multiple generations.

**A3 1:** Dynamics of a neutral allele's frequency in a haplodiploid population

Generation	Frequency in females (*p*_f_)	Frequency in males (*p*_m_)	‘*Per capita*’ frequency (*p* ≈ *p*_f_)	RV-weighted frequency (*p*^*^ = *c*_f_*p*_f_ + *c*_m_*p*_m_)
1	0.0000	1.0000	0.0000	0.3333
2	0.5000	0.0000	0.5000	0.3333
3	0.2500	0.5000	0.2500	0.3333
4	0.3750	0.2500	0.3750	0.3333
5	0.3125	0.3750	0.3125	0.3333
6	0.3438	0.3125	0.3438	0.3333
7	0.3281	0.3438	0.3281	0.3333
8	0.3359	0.3281	0.3359	0.3333
9	0.3320	0.3359	0.3320	0.3333
10	0.3340	0.3320	0.3340	0.3333

The *per capita* frequencies are plotted in Figure 3 panel (a). Note that the frequency of the A allele in females asymptotes to *p* = 1/3 (the same is true of its frequency in males). Thus, there is an apparent long-term increase in allele A's frequency of (1/3) – 0 = 1/3. Both alleles are neutral, so this is not the work of natural selection. Rather, it is a class effect. The class reproductive value of males is *c*_m_ = 1/3 under haplodiploidy, which means that 1/3 of genes in the distant future trace back to males and *c*_f_ = 2/3 trace back to females, under neutrality. Awarding each of the *n*_m_ males in the population, an equal share of their class's reproductive value yields a male's reproductive value of *f*_m_ = 1/(3*n*_m_). Similarly, the reproductive value of each female is *f*_f_ = 2/(3*n*_f_), where *n*_f_ is the number of females in the population. Because *n*_f_ ≫ *n*_m_, then *f*_m_ ≫ *f*_f_; that is, an individual male has higher reproductive value than an individual female, and so the A allele – which is overrepresented in males – enjoys an increase in frequency owing to the class effect.

Panel (b) reveals the fate of the A allele if it enjoys a selective advantage of 100% in generation 1 (solid line) and thereafter behaves neutrally (broken line). This makes no difference to its course over the generations. This is because there is no genetic variance within either class in generation 1 and, hence, there is no selection operating within either class in this generation. Panel (c) reveals the fate of the allele if it enjoys its selective advantage in generations 1 and 2 (solid line) and thereafter behaves neutrally (broken line). In generation 2, there is genetic variation among females, and consequently, the A allele is favoured by natural selection in this generation. Note that its actual frequency decreases from generation 2 to generation 3, but less sharply than it would have done under neutrality (grey broken line). This is reflected in its asymptotic frequency being > 1/3, and this disparity Δ_2_ in its asymptotic frequency defines the selective progress it made on the account of the fitness superiority of its bearers in generation 2. Panel (d) reveals the fate of the A allele if it enjoys a selective advantage over 10 generations: it rises towards fixation. Its selective progress in each generation can be measured by contrasting with counterfactuals in which it was neutral in this and every subsequent generation (grey broken lines). The selective progress attained in generations 2, 3, 4 and 5 is indicated by arrows.

Also included in Table1 are reproductive-value-weighted allele frequencies, *p** = *c*_f_*p*_f_ + *c*_m_*p*_m_. These describe the average frequency in males and females, weighting each sex's allele frequency by its reproductive value. The calculation for generation 1 is (2/3) × 0 + (1/3) × 1 = 1/3; in generation 2, the calculation is (2/3) × (1/2) + (1/3) × 0 = 1/3; and the frequency remains at *p** = 1/3 for every subsequent generation. Thus, weighting each class's allele frequency by its reproductive value when calculating the population frequency of the allele provides an alternative – but equivalent – means for removing the class effect from allele frequency change (Fisher, 1930; Lehmann & Rousset, 2014), as discussed in Appendix 2.

This neutrality scenario is plotted in Figure4 panel (a). And panel (b) again reveals the fate of the A allele if it enjoys a selective advantage of 100% in generation 1 (solid line) and thereafter behaves neutrally (broken line): there is no change in the allele's frequency, because there is no response to natural selection (as there is no genetic variation within either class) in generation 1. Panel (c) again reveals the fate of the allele if it enjoys its selective advantage in generations 1 and 2 (solid line) and thereafter behaves neutrally (broken line). In generation 2, there is genetic variation among females, and consequently, the A allele is favoured by selection. It increases in frequency in this generation only and thereafter remains at its new frequency. Note that this increase in frequency Δ_2_ is exactly equal to the asymptotic progress made by the allele in Figure3 panel (c). Thus, the reproductive value weighting recovers the asymptotic fate of the allele, but describes this effect immediately in the generation in which selection has operated. That is, natural selection acting in the present generation has gene frequency consequences for the long-term future, and reproductive value weightings provide a means for describing these future consequences immediately in the present. Panel (d) illustrates this principle for multiple generations of selection.
